# The Role of Uncertainty Mindsets in Shaping Diversity Attitudes and Their Downstream Effects on Commitment to Societal Change and Right-Wing Populist Voting

**DOI:** 10.1177/01461672251386487

**Published:** 2025-11-11

**Authors:** Ruri Takizawa, Stefanie Marx-Fleck, Alina Gerlach, Gudela Grote

**Affiliations:** 1 ETH Zurich, Switzerland; 2 Goethe University Frankfurt, Germany

**Keywords:** diversity attitudes, uncertainty mindset, intervention, social change, right-wing populism

## Abstract

How people respond to uncertainty has far-reaching socio-political consequences. When perceived as fixed and threatening, individuals may gravitate toward ideologies that undermine intergroup relations and social cohesion. We examined whether fostering an *uncertainty-as-enabling* mindset (viewing uncertainty as malleable and an opportunity) promotes positive diversity attitudes and indirectly affects commitment to societal change and support for right-wing populism. During Germany’s 2025 federal election, we ran a 3-month online study with German citizens (*N* = 745). Participants who received an uncertainty mindset intervention (vs. control) reported a higher uncertainty-as-enabling mindset, which was linked to stronger commitment to societal change through higher perceived productivity of diversity and lower aversion to intergroup contact. The mindset was also linked to a lower likelihood of voting for a right-wing populist party via the same pathways. Shifting how individuals appraise uncertainty may thus be a promising strategy for strengthening democratic resilience by cultivating openness to diversity.

## Introduction

“Young and radical: Why right-wing populism is on the rise” (von Nahmen, 2025) and “Rightwing populists will keep winning until we grasp this truth about human nature” (Monbiot, 2025) are two examples of headlines that capture growing concern over political trends in Western democracies. Right-wing populist parties typically promote an ideological constellation of nativism, authoritarianism, and populism (Mudde, 2007), reflecting a rejection of social change toward equality and undermining fundamental democratic principles, such as pluralism and the protection of minority rights (Meléndez & Kaltwasser, 2023; Mols & Jetten, 2020). In recent years, rising political uncertainty has coincided with growing support for anti-diversity ideologies and right-wing populist parties, which pose challenges to social equality (Aktas, 2024; Mudde, 2019).

These political shifts reflect both individual and collective responses to perceived threats, that is, situations that undermine the stability, identity, and continuity of groups and societies (Stollberg & Jonas, 2021). Prior research shows that such an experience of uncertainty heightens people’s need for personal security and order, while triggering concerns about the vitality and distinctiveness of their social group (Jetten & Wohl, 2011; Wohl et al., 2010). Individuals may thus lean toward conservative and authoritarian ideologies that prioritize the preservation of traditional social hierarchies (Bonanno & Jost, 2006; Hogg et al., 2013). As a result, they may exhibit more bias toward outgroup members, oppose diversity-enhancing policies, and show more support for right-wing populist parties (Craig & Richeson, 2014; Gründl & Aichholzer, 2020; Major et al., 2016). From this perspective, the reported political shifts can be understood as individuals’ psychological attempts to cope with uncertainty (Park & Folkman, 1997). However, such responses often come at the cost of positive intergroup relations, particularly by exacerbating the marginalization of minority group members. Moreover, uncertainty, defined as a state of “not knowing for sure” (Grote, 2009; Hogg, 2000), is not merely a temporary disruption but a fundamental and enduring condition of human life. This raises a critical question: What kind of approaches to uncertainty can buffer against the polarizing, defensive, or exclusionary responses to uncertainty that are aggravated by political instability, demographic change, or perceived cultural erosion?

Grounding our study in a highly polarized election period, we illuminate the mechanisms that influence whether people respond to perceived threats with openness to social transformation (i.e., commitment to societal change) or with retrenchment and rejection (i.e., voting for right-wing populist parties). We examined whether we could indirectly promote the former and reduce the latter by shaping how individuals interpret and respond to uncertainty. Drawing on recent research suggesting that people can conceptualize uncertainty as malleable (vs. fixed) and as an opportunity (vs. a threat; Griffin & Grote, 2020; Magni et al., 2025), we examined whether adopting an *uncertainty-as-enabling* mindset predicts greater recognition of diversity’s positive aspects, less aversion to intergroup contact, and, in turn, stronger commitment to societal change and a lower likelihood of voting for right-wing populist parties.

### Uncertainty and Political Extremism

When uncertainty is perceived merely as a negative feature, people tend to avoid or minimize it (FeldmanHall & Shenhav, 2019; Hogg, 2007), even when doing so incurs personal costs (see Alquist & Baumeister, 2024). This tendency is evident not only at the individual but also at the societal level: For instance, Hofstede’s (1984) dimension of *uncertainty avoidance* reflects a cultural preference for clear rules to cope with ambiguity. Higher uncertainty avoidance has been linked to conservatism, resistance to change, and lower tolerance for diversity (e.g., Leong & Ward, 2006). Such insights illustrate the pervasiveness of uncertainty avoidance across levels of analysis.

At the individual level, social psychological theories specify cognitive and motivational processes that explain how uncertainty can undermine intergroup relations and drive individuals toward extremist movements (Hogg, 2000, 2007; Jost, 2019). Uncertainty heightens anxiety, both generally and in relation to one’s group’s perceived social status, which in turn increases epistemic needs for closure, order, and defending the status quo. In seeking to fulfill these needs, people may resort to cognitive shortcuts and heuristics, such as stereotyping and essentialist thinking (Hogg et al., 2013), which have been linked to stronger endorsement of conservative political beliefs (Eidelman et al., 2012; Van Berkel et al., 2015) and authoritarian ideologies (Jost et al., 2003). Such ideologies are associated with maintaining social hierarchies and resisting egalitarian change.

Specific group affiliations can also serve as a platform for individuals to affirm their social status and enact ideological beliefs outlined above. The literature suggests that under self-uncertainty, individuals are drawn to groups that provide rigid boundaries and norms (Hogg, 2007). Extremist groups that emphasize a strong distinction between “us” and “them” offer psychological benefits, including predictability and a sense of belonging (Hogg & Adelman, 2013). Right-wing populist movements fit this pattern: they combine hierarchical group structures with clearly defined ingroup–outgroup distinctions (Gründl & Aichholzer, 2020). While such group memberships may initially offer a sense of psychological security, they may ultimately erode social cohesion or democratic principles (Hogg, 2024). Thus, perceiving uncertainty as fixed and as a threat can harm intergroup relations and inhibit support for societal changes that benefit disadvantaged groups.

To address these tendencies, researchers have focused on interventions designed to *reduce uncertainty* in intergroup interactions. Efforts to improve intergroup relations bring members of conflicting groups into direct, indirect, or imagined contact across diverse contexts (Čehajić-Clancy & Halperin, 2024; Zhou et al., 2019). While these interventions can be effective when carefully tailored to the specific individuals and groups involved, they may also backfire, for example, when participants adopt a prevention-focused approach (Guffler & Wagner, 2017; West & Greenland, 2016). Crucially, however, this work largely overlooks approaches that intentionally embrace or even cultivate uncertainty as a means of promoting more adaptive, opportunity-focused responses.

### Uncertainty Appraisal

The focus on uncertainty reduction reflects psychology’s prevailing bias toward avoidance rather than recognizing its potential benefits, and this narrow focus may be insufficient for fully understanding human behavior (Alquist & Baumeister, 2024). Newer theories offer a nuanced explanation of how people navigate uncertainty in everyday life: the uncertainty regulation theory advocates for a more balanced view of uncertainty *regulation*, rather than solely considering uncertainty reduction (Griffin & Grote, 2020). It proposes that individuals regulate uncertainty dynamically, engaging in both opening and closing behaviors to align their preferred level of uncertainty with situational demands. These behaviors reflect principles of psychological self-regulation and the exploration–exploitation trade-off (Gershman, 2019): opening involves seeking novelty, new experiences, or social contexts (exploration), thereby increasing uncertainty, while closing relies on familiar routines, established knowledge, and predictable outcomes (exploitation) to reduce it. Exploration and exploitation behaviors have been applied across various domains (e.g., Tsang et al., 2025).

Empirical studies examined uncertainty appraisal using a *mindset approach* (Magni et al., 2025). A mindset reflects beliefs by which individuals process information, and therefore has an impact on how the world is perceived and how someone acts (Dweck, 2006; Dweck & Yeager, 2020). Prior research has investigated how different mindsets influence cognitive, affective, and physiological responses. For example, people’s tendency to exhibit a growth mindset about intelligence increases their effort in learning and task performance (Burnette et al., 2012, 2023), and a stress mindset predicts how people interpret their experiences of stress (Crum et al., 2013). Unlike stable individual traits, which, in the context of uncertainty, are often conceptualized as dispositions to view uncertainty as a discomfort to be avoided (Gerlach & Pfrombeck, 2025), mindsets are more malleable and receptive to change. Recent research has conceptualized that individuals can form beliefs about uncertainty in the form of an uncertainty mindset. A higher uncertainty-as-enabling mindset is characterized by viewing uncertainty as malleable and an opportunity, which has been associated with greater exploration tendencies, in contrast to an uncertainty-as-disabling mindset, where one views uncertainty as fixed and a threat (Magni et al., 2025).

To date, little work has explored the role of mindsets in shaping intergroup or diversity-related attitudes. We argue that studying the uncertainty mindset is particularly valuable in this context. Existing evidence suggests that beliefs characterized by flexibility, rather than rigidity, are generally associated with more positive outgroup emotions, attitudes, and behavioral intentions (Halperin et al., 2012; Hoyt et al., 2024). This pattern aligns with cognitive research indicating that inflexibility predicts extremist political attitudes (Zmigrod et al., 2019). An uncertainty-as-enabling mindset reflects cognitive flexibility by highlighting the benefits and opportunities of uncertainty. Therefore, when individuals consciously adopt this mindset, they should be more likely to embrace the uncertainty inherent in diversity, an attitude that is associated with openness to societal transformation, rather than rigidly clinging to the familiar or seeking control and status through outgroup bias and support for extremist groups.

### Uncertainty Mindset Intervention

Conceptualizing uncertainty appraisal in the form of an uncertainty mindset opens the door for change (Walton & Crum, 2021). Wise interventions aim to induce specific and lasting changes in complex systems that entail interactions of the person and the situation (Walton & Wilson, 2018). For example, growth and stress mindsets have been shown to be malleable through brief, scalable interventions when they are carefully designed and implemented (Crum et al., 2023; Paunesku et al., 2015). Preliminary evidence suggests that uncertainty mindsets are also changeable (Strittmatter et al., 2024). For example, an uncertainty mindset intervention implemented with unemployed individuals led to less anxiety in job search, more self-efficacy, and perceptions of occupational opportunities (Pfrombeck et al., 2024). By reframing uncertainty as malleable and an opportunity, interventions can foster greater openness to exploration behaviors across various domains. However, the uncertainty mindset and its related perspective of uncertainty as enabling have yet to be investigated in relation to intergroup attitudes and behaviors. Given the role of uncertainty in shaping intergroup dynamics and political polarization, this represents a critical gap and an opportunity.

### The Present Research

In times of heightened uncertainty, individuals may be drawn toward anti-diversity ideologies, adherence to traditional social hierarchies, and extremist political groups. Interventions that help individuals reframe uncertainty from fixed and a threat into malleable and an opportunity may reduce such tendencies by promoting exploration over exploitation (Alquist & Baumeister, 2024; Griffin & Grote, 2020; Hogg, 2024; Magni et al., 2025). From a social psychology perspective, exploration tendencies correspond to challenging traditional social, economic, and political systems, and an openness to social change, while exploitation tendencies relate to defending social hierarchies and maintaining the status quo (e.g., Jost, 2019). In this study, we examined whether helping individuals better regulate uncertainty (rather than simply trying to minimize it) could increase their openness to diversity, which is linked to socio-political consequences. We introduced a general uncertainty mindset intervention and investigated whether cultivating an uncertainty-as-enabling mindset is indirectly linked to two key outcomes: (a) higher commitment to societal change (i.e., openness to departing from the status quo) and (b) lower support for a political party characterized by right-wing populist tendencies.

The study was conducted during Germany’s 21st federal election cycle in 2025 using a between-participants experimental design. Data collection spanned six measurement periods from mid-December 2024 to early March 2025. An overview of major national socio-political events reported during this time, based on coverage from *Tagesschau*, Germany’s most widely viewed news television program (Norddeutscher Rundfunk, 2025), is provided in the Supplemental Material (SM1). Immigration, refugee policy, and the social integration of minority groups were central issues in the election. Originally scheduled for September, the election was moved to February following a government crisis that led to the collapse of the ruling coalition. This shift in the electoral timeline occurred in a broader climate of political uncertainty, which was widely noted in both national and international discourses (Kreilinger, 2025; McGuinness, 2024).

Grounded in the literature of uncertainty regulation theory (Griffin & Grote, 2020), mindsets (Dweck, 2006; Hoyt et al., 2024; Magni et al., 2025), and wise interventions (Walton & Wilson, 2018), we proposed that a brief uncertainty mindset intervention (Strittmatter et al., 2024) shifts how individuals appraise uncertainty. Considering theoretical and initial empirical evidence that an uncertainty-as-enabling mindset predicts exploration tendencies, we hypothesized that an uncertainty-as-enabling mindset will be associated with more positive diversity attitudes, manifested in a stronger belief in the productivity of diverse groups and lower aversion to interacting with individuals from other social groups. Recognizing the benefits of living in a diverse society this way aligns with a more inclusive worldview that counters threat-based rigidity and resistance to change (Hogg, 2024; Jost, 2019). Therefore, we expected that positive diversity attitudes are associated with more commitment to societal change and a lower likelihood of voting for a right-wing populist party (Hogg & Adelman, 2013). Thus, our hypotheses were the following (see Figure 1):

**Hypothesis 1 (Uncertainty Mindset → Diversity Attitudes):** A higher uncertainty-as-enabling mindset will be associated with (a) a stronger belief in the productivity of diverse groups and (b) lower aversion to intergroup contact.
**Hypothesis 2 (Uncertainty Mindset → Diversity Attitudes → Commitment to Societal Change):** A higher uncertainty-as-enabling mindset will be associated with (a) a stronger belief in the productivity of diverse groups and (b) lower aversion to intergroup contact, which are both linked to a stronger commitment to societal change.
**Hypothesis 3 (Uncertainty Mindset → Diversity Attitudes → Right-wing Populist Party Vote):** A higher uncertainty-as-enabling mindset will be associated with (a) a stronger belief in the productivity of diverse groups and (b) lower aversion to intergroup contact, which are both linked to a lower likelihood of voting for a right-wing populist party.

**Figure 1.**
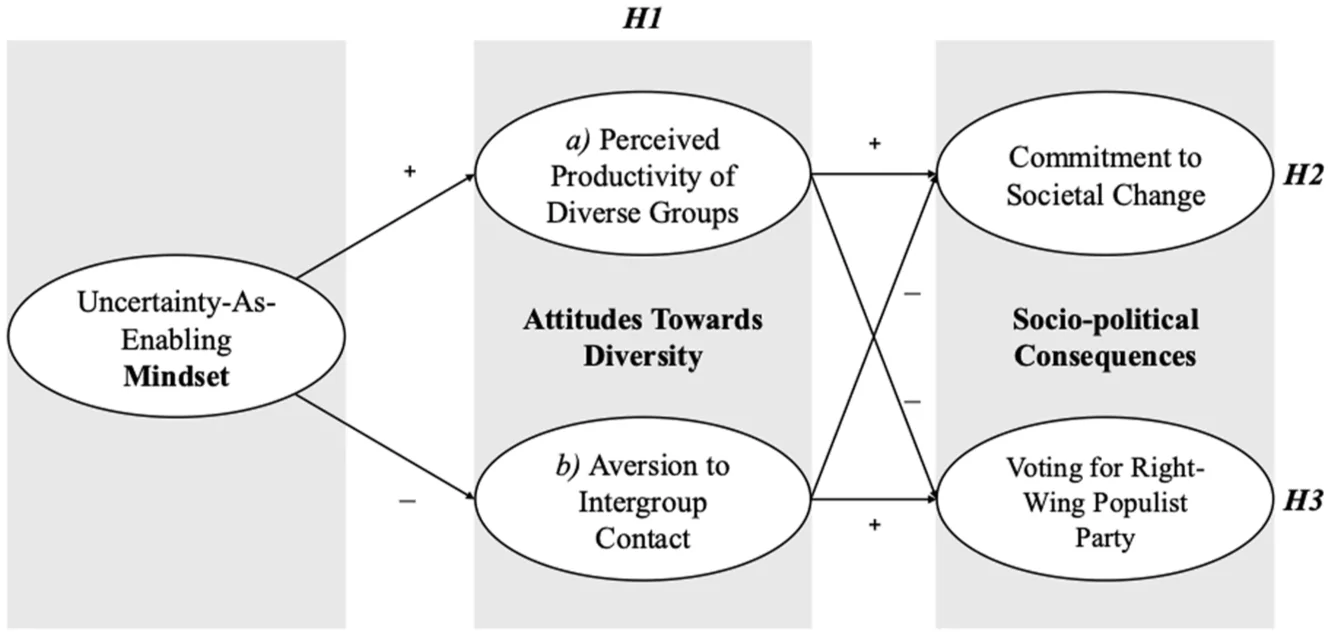
Hypothesized Conceptual Model.

## Method

### Transparency and Openness

The research was approved by the ETH Zurich Ethics Commission (Project 24 ETHICS-379). We report all recruitment decisions, data exclusions, manipulations, and measures. Any measures not used for hypothesis testing are fully described in SM2. All data, analysis code, codebook, and research materials are available at http://osf.io/9ks2n. Data were analyzed using IBM SPSS Statistics, version 29.0.2.0 (20), R, version 4.5.0 (R Core Team, 2025), and the packages Lavaan, version 0.6.19 (Rosseel, 2012), and haven, version 2.5.4 (Wickham et al., 2023). This study was not pre-registered.

### Participants

Data were collected in six waves (T1–T6), timed around the German federal election held on February 23, 2025. We initially conducted a Monte Carlo power analysis with the Shiny app^1^ (Schoemann et al., 2017) to estimate the sample size needed to detect our two hypothesized indirect effects (H2a-b) on commitment to societal change (our continuous outcome). Based on prior work showing small to moderate effects of mindsets on attitudes and support for social change (e.g., Burnette et al., 2012; Crum et al., 2013; Vani et al., 2023), we set all standardized paths to β = .20 (*SD* = 1.0). Simulations (1,000 replications, 200 draws per replication; seed = 1,234; α = .05, two-tailed; target power = .90) indicated that, for *N* = 500 to 800, power to detect each indirect effect ranged from .93 to .99. Thus, to ensure adequate power despite attrition, we contracted Bilendi (https://www.bilendi.de/) to recruit a panel of 1,000 German nationals living in Germany at T1. Quotas for age group and gender were used to align the sample with population benchmarks. Participants provided informed consent, passed an initial attention check, and received compensation in accordance with Bilendi’s standard rates. To bolster retention, three randomly selected respondents who completed all six surveys were additionally awarded a €50 bonus.

The final sample consisted of 391 participants in the intervention group and 354 in the control group, resulting in an overall attrition rate of 31.4%. Participants were 18 to 80 years old (*M* = 46.63, *SD* = 15.23; 50.1% women and 49.9% men^2^); 34.5% held a tertiary education degree. In terms of employment, 52.3% worked full-time, 18.7% part-time, and 5.0% were unemployed. To assess socioeconomic status, we asked participants to rate their household income relative to the 2024 median net income in Germany (€35,510; Statista, 2024) from 1 *very much above* to 7 *very much below* (*M* = 3.90, *SD* = 1.71). Participants’ subjective social status was assessed with the 10-rung MacArthur ladder (Adler et al., 2000; *M* = 5.55, *SD* = 1.73). All 16 German federal states were represented. The largest cohorts lived in North Rhine-Westphalia (22.1%), Bavaria (16.8%), and Baden-Wurttemberg (10.6%). On a 5-point scale of residential context (1 *very rural* to 5 *very urban*), participants’ ratings averaged 3.28 (*SD* = 1.20).

### Procedure

Participants received an email invitation with a link to the Qualtrics questionnaire to participate in the “study on attitudes in society.” In T1 (December 16–23, 2024), participants were randomly assigned to either the intervention or control condition when they accessed the questionnaire and answered socio-demographic questions. Only participants in the intervention condition were invited to the questionnaire in T2 (January 6–13, 2025). All participants filled out the same questionnaires at T3 (January 13–20, 2025), T4 (January 20–27, 2025), and T5 (February 10–17, 2025), which included the uncertainty mindset scale (Magni et al., 2025) and the measure to capture attitudes toward diversity (Nakui et al., 2011). After the elections on February 23, participants filled out the final questionnaire in T6 (February 24–March 3, 2025), which assessed their commitment to societal change and voting behavior. After being debriefed about the study goals and experimental conditions, participants could indicate if they wanted their data deleted. Figure 2 provides a detailed overview of dropouts and exclusions.

**Figure 2.**
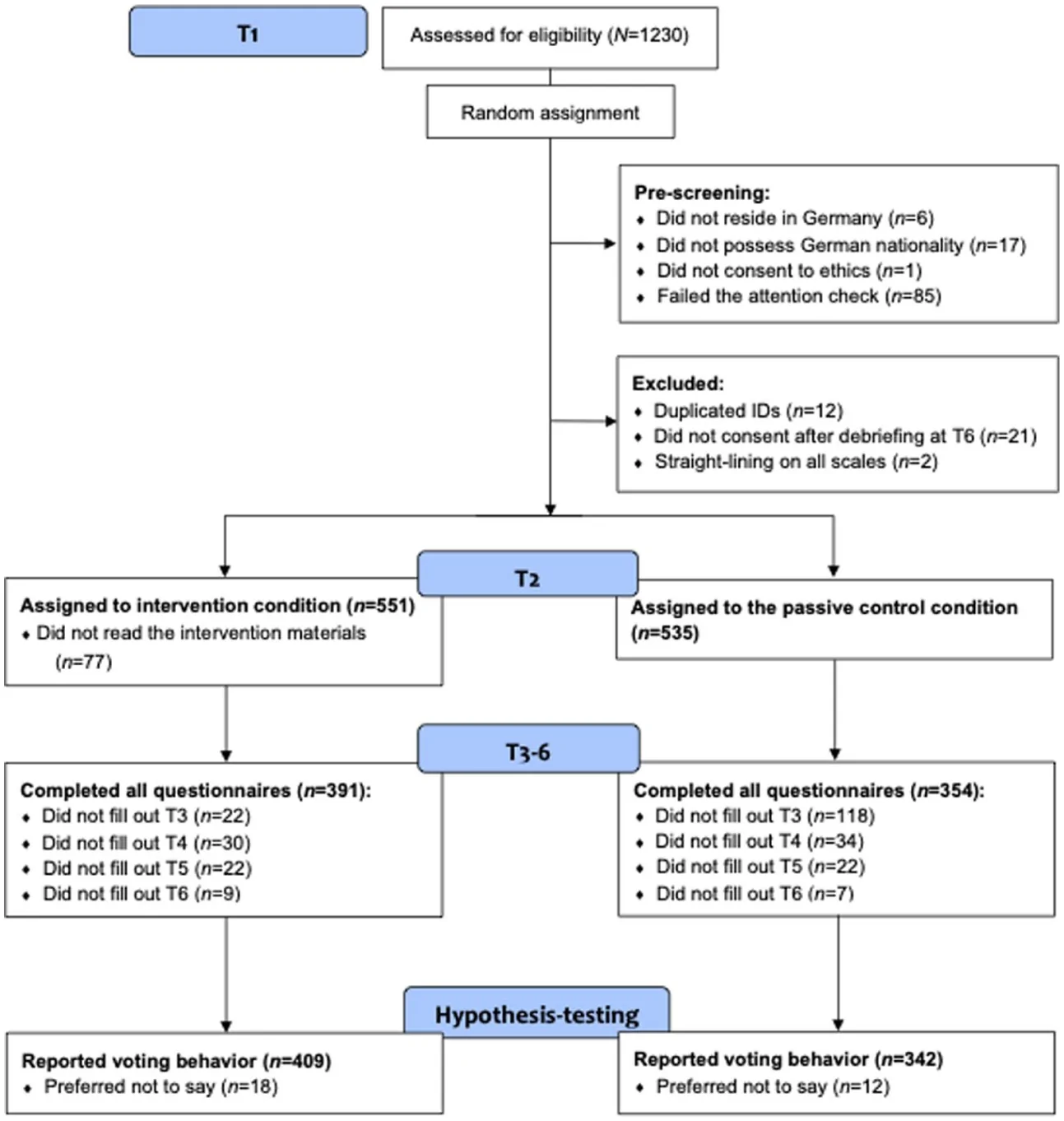
Flow of Participants Through Each Stage of the Study.

### Mindset Intervention

We adapted our intervention based on materials used in previous studies (Pfrombeck et al., 2024; Strittmatter et al., 2024). Two pilot studies were conducted via Prolific with native German speakers residing in Germany (*N*_Pilot1_ = 68, *N*_Pilot2_ = 32). We collected quantitative and qualitative feedback on credibility, comprehension, relevance, and design elements, which informed iterative refinements to the materials. The final intervention consisted of 11 slides that combined text, graphics, and photographs. The material defined uncertainty as a state of not knowing something for sure and emphasized its ubiquity in everyday life. It acknowledged that although uncertainty can be unpleasant, it is often manageable through one’s actions. The presentation conveyed that uncertainty could foster personal growth and help individuals reach their full potential. Several slides presented scientific findings on the beneficial effects of uncertainty, citing studies on memory, creativity, and affect. Finally, the slides highlighted Steve Jobs as a well-known example of someone whose decision to pursue an uncertain path in life led to extraordinary opportunities. We included short excerpts from his 2005 Stanford University commencement address, which is widely regarded for its lasting inspiration (e.g., “I decided to drop out and trust that it would all work out OK. It was pretty scary at the time, but looking back it was one of the best decisions I ever made”). Following the presentation, participants engaged in a structured reflection task. First, they were asked to imagine a friend, family member, or colleague and compose a brief note explaining what they had just learned about uncertainty. Next, participants reflected on their own recent experience of a situation that contained uncertainty. They wrote a short message describing the situation and how they might now interpret or reframe it based on the information presented. The median time to complete the intervention was 7.5 min.

For exploratory purposes, we systematically analyzed participants’ written reflections on their personal experiences with uncertainty. Two independent coders reviewed the responses and interpreted the content to categorize the context of uncertainty. The initial interrater agreement was 77.9%, and after the discussion, the coders reached a full consensus on all categorizations. The identified topics were education, finance, health/personal safety, housekeeping/household, relationships, job/career, move, politics, travel, and future in general. The most frequently mentioned themes were job/career, followed by health/personal safety, relationships, and finance.

### Measures

Unless otherwise indicated, 7-point Likert-type scales were used, with higher scores indicating agreement.

#### Uncertainty Mindset at T3-T5

We administered the German version of the Uncertainty Mindset Scale, comprising 22 items (Magni et al., 2025). The mindset consists of four subdimensions, which capture the extent to which uncertainty is perceived as fixed (e.g., “I believe I cannot change the uncertainty that I experience”), malleable (e.g., “If I collect more information, I can change the amount of uncertainty”), a threat (e.g., “Uncertainty in life is a threat to the achievement of my life goals”), or an opportunity (e.g., “Uncertainty in life creates opportunities”). We treated it as an overarching uncertainty-as-enabling construct (*M*_T3_ = 4.55, *SD*_T3_ = 0.87; *M_T4_* = 4.56, *SD_T4_* = 0.89; *M_T5_* = 4.56, *SD_T5_* = 0.92), with higher scores indicating that individuals view uncertainty more as malleable and as an opportunity, and less as fixed (reverse-coded) and as a threat (reverse-coded). Reliability of the scale was high (Cronbach’s α_T3-T5_ = .90–.91).

#### Attitudes Toward Diverse Groups in Society at T4-T5

To capture people’s attitudes about living and interacting with people and groups that vary in diversity, we adapted Nakui et al.’s (2011) Attitudes Toward Diverse Workgroups Scale, which originally assessed attitudes toward diversity in work settings and was reworded to capture the experience with diverse groups more generally. Participants read,

Today, people are often confronted with groups that vary in their diversity in terms of age, gender, ethnicity, expertise, background, and country of origin. We would like to know your feelings about interacting with groups that vary in their degree of diversity. A diverse group is one in which people differ from one another in one or more significant ways.

As in the original, we examined two dimensions: perceived productivity of diversity (11 items; for example, “Talking to people from diverse groups can increase one’s understanding of those who are different from me”) and aversion to intergroup contact (six items; for example, “I don’t enjoy interacting with people who come from different countries”). All items were translated into German using a back-translation procedure. Both subscales demonstrated high internal consistency across waves (productivity α_T4-T5_ = .95–.96; aversion α_T4-T5_ = .86). Higher scores reflect stronger beliefs in the productivity of diverse groups (*M*_T4_ = 4.61, *SD*_T4_ = 1.25; *M*_T5_ = 4.60, *SD*_T5_ = 1.32) and greater aversion to interacting with diverse others (*M*_T4_ = 4.20, *SD*_T4_ = 1.21; *M*_T5_ = 4.30, *SD*_T5_ = 1.23).

#### Commitment to Societal Change at T6

To capture commitment to societal change, we adapted the affective component of the commitment to change scale (Herscovitch & Meyer, 2002), which was originally developed for organizational settings. The scale was translated into German using a back-translation procedure. The measure comprised six items (e.g., “Societal change leads to positive developments for Germany”), with higher values indicating stronger commitment to societal change (α = .89; *M* = 4.67, *SD* = 1.26). The scale items were designed to be neutral with respect to the direction of change (progressive vs. reactionary; Becker, 2020) to reduce the risk of participant dropout due to perception of political bias or the activation of demand characteristics.

#### Right-Wing Populist Voting Behavior at T6

Participants were asked to report their voting behavior in the 2025 German federal election (second vote; see Table 1). The sample’s reported voting behavior closely aligns with the actual electoral results (The Federal Returning Officer, 2025).

**Table 1.** Distribution of Participants’ Self-Reported Voting Behavior in the 2025 Federal Election.

Party	Count	%
AfD (Alternative for Germany)	150	20.1
BSW (Sahra Wagenknecht Alliance)	49	6.6
CDU/CSU (Christian Democratic Union)	170	22.8
DIE LINKE (The Left)	59	7.9
FDP (Free Democratic Party)	35	4.7
GRÜNE (The Greens)	74	9.9
SPD (Social Democratic Party)	119	16.0
SSW (South Schleswig Voters’ Association)	2	0.3
Different party	26	3.5
Do not want to indicate	30	4.0
Did not vote	31	4.2

*Note.* Ten participants who voted for a “different party” wrote to have voted for the following parties: Die Partei, Bündnis Deutschland, Freie Wähler, Bündnis C, Bündnis Deutschland, Volt, Partei Mensch Umwelt Tierschutz, and die deutsche Mitte.

For testing H3, we categorized participants into two groups: those who voted for a party classified as right-wing on social policy (restrictive positions on migration and an emphasis on assimilation in integration policy), *and* marked by populist framing of society (casting “the people” against a perceived corrupt elite) versus those who did not vote for such a party (Craig & Richeson, 2014; Gründl & Aichholzer, 2020; Major et al., 2016; Mudde, 2019). Regarding the first criterion, three parties advocated for more restrictive immigration policies and emphasized assimilation (Thomeczek et al., 2024). These included the Alternative for Germany (*Alternative für Deutschland*, AfD), the newly founded left-wing nationalist party Sahra Wagenknecht Alliance (*Bündnis Sahra Wagenknecht*, BSW), and the center-right Christian Democrats (CDU/CSU), all of which called for significant reforms to Germany’s immigration and asylum systems (Deutschlandfunk, 2025). Among these parties, the AfD could be most clearly classified as a right-wing populist party (Arzheimer & Berning, 2019; Lees, 2018; Mudde, 2019). Founded in 2013, the AfD initially focused on dissolving the Eurozone and restructuring German foreign policy (Arzheimer, 2015). However, it soon adopted a more populist orientation, centering its rhetoric on immigration and refugee policy, and contributed to the polarization of political discourse (Atzpodien, 2022). To test our model, support for right-wing populism was operationalized as participants’ likelihood of voting for the AfD. For H3, we compared those who had voted for the AfD (*n* = 150) with those who had not (*n* = 565). Participants who chose not to disclose their voting behavior (*n* = 30) were excluded from this analysis.

## Analytic Strategy and Results

Table 2 contains the intercorrelations among socio-demographic and primary variables for hypothesis testing.

**Table 2.** Pearson’s Correlations Between Socio-Demographic Variables at T1, Uncertainty Mindset at T3, Diversity Attitudes at T4, and Political Measures at T6.

Variable	Age (T1)	1	2	3	4	5	6	7	8	9	10
Female gender (T1)	.05	**—**									
Full-time employment (T1)	–.32***	–.26***	—								
Completed tertiary education (T1)	–.21***	–.08*	.18***	—							
Objective social status (T1) (*higher score = below average*)	.17***	.12***	–.43***	–.22**	**—**						
Subjective social status (T1) (*higher score = higher status*)	–.15***	–.02	.28***	.28***	–.59***	**—**					
Residential area (T1) *(higher score = very urban)*	.00	–.07	–.04	.08*	.11**	–.03	**—**				
Uncertainty mindset (T3)	.16***	.02	–.03	.01	–.11**	.20**	.04	**—**			
Perceived productivity of diversity (T4)	–.02	.07	.03	.15***	–.11**	.20***	.13***	.25**	**—**		
Aversion to intergroup contact (T4)	–.09*	.08*	.02	–.07	.04	–.05	–.06	–.17***	–.44***	**—**	
Commitment to societal change (T6)	.04	.06	–.06	.09*	–.03	.11**	.13***	.26***	.47***	–.29***	**—**
Voted for AfD (T6)	–.01	–.05	.07	–.11**	.03	–.14***	–.10**	–.00	–.36***	.25***	–.24***

*Note. N* = 715. T_i_ = study wave; AfD = Alternative für Deutschland (Alternative for Germany).

* *p* < .05. **p < .01, ***p < .001.

### Preliminary Analysis

We first examined whether the intervention influenced participants’ uncertainty mindset across the three post-intervention measurement points (T3–T5) using a linear mixed-effects model with repeated measures nested within participants. Time was modeled as a numeric covariate centered on the middle occasion (–1 = T3, 0 = T4, and 1 = T5). The model included fixed effects of time, condition (0 = control and 1 = intervention), and their interaction, as well as random intercepts and uncorrelated random slopes for time at the participant level (restricted maximum likelihood [REML]). The model explained 72.1% of the total variance in the uncertainty mindset and indicated high between-person stability (intraclass correlation coefficient [ICC] = .72). We found a main effect of the intervention, *F*(1, 743.80) = 5.25, *p* = .022. At the centered time point (T4), the model-estimated intervention-control difference was 0.13 (95% confidence interval [CI] = [0.02, 0.25]), such that participants who received the intervention reported a higher uncertainty-as-enabling mindset than those in the control condition. The linear effect of time was non-significant, *F*(1, 741.48) = 0.01, *p* = .929, indicating no general change in mindset across time points. The interaction between time and intervention was also not significant, *F*(1, 741.48) = 0.40, *p* = .527. Thus, the intervention effect was stable across T3 to T5. Random effects showed between-person variability in intercepts (0.57, *SE* = .03), but negligible variability in linear time slopes (≈ 0.001, *SE* = .01) and residual variance (σ^2^ = 0.22), suggesting little person-specific linear change over time.

### Measurement Models (CFA)

To establish construct validity and empirical distinctiveness of our four focal latent variables (see Figure 1), we ran a series of confirmatory factor analysis (CFA) models using robust maximum likelihood with full-information maximum likelihood for missing data. The models included the uncertainty-as-enabling mindset (specified as a second-order factor reflected by four first-order subdimensions) from T3, diversity attitudes (productivity and aversion) from T4, and commitment to societal change from T6. Because the voting outcome was categorical, it could not be modeled as a latent variable. We compared three nested models: (a) our hypothesized four-factor model, χ^2^(935) = 3,086.54, *p* < .001, comparative fit index (CFI) = .90, Tucker–Lewis index (TLI) = .89, root mean square error of approximation (RMSEA) = .06, 90% CI = [0.05, 0.06], standardized root mean square residual (SRMR) = .08; (b) a three-factor model that collapsed diversity attitudes into a single latent construct, χ^2^(697) = 3,518.55, *p* < .001, CFI = .84, TLI = .83, RMSEA = .07, 90% CI = [0.07, 0.08], SRMR = .08; and (c) a one-factor model in which all items loaded onto a single factor, χ^2^(945) = 13,545.51, *p* < .001, CFI = .40, TLI = .37, RMSEA = .13, 90% CI = [0.13, 0.14], SRMR = .16. The four-factor model demonstrated the best fit and outperformed both alternatives: Δχ^2^(238) = 432.01, *p* < .001 (vs. three factors) and Δχ^2^(10) = 10,458.97, *p* < .001 (vs. one factor). As a robustness check, we repeated the comparison using the diversity attitude measures from T5, which yielded an almost identical pattern (SM4).

### Hypothesis Testing (SEM)

To test our hypotheses, we specified a structural equation model (SEM) using robust weighted least squares mean and variance adjusted (WLSMV) estimation (with the ordered argument for the binary voting outcome). Considering our experimental design, we treated the uncertainty mindset intervention as an independent variable in our mediation model because it was expected to exert a direct effect on the uncertainty mindset but not on downstream outcomes. We then applied serial mediation to test our hypothesized pathways (H1–H3). Thus, in accordance with our theoretical framework, the model included the T2 intervention (0 = *control* and 1 = *intervention*) predicting the uncertainty-as-enabling mindset at T3, which in turn predicted perceived productivity of diverse groups and aversion to intergroup contact at T4. Both mediators and the uncertainty-as-enabling mindset then predicted the likelihood of voting for a right-wing populist party (1 = *voted for AfD* and 0 = *did not*) and commitment to societal change (continuous) at T6. We applied listwise deletion (one participant), yielding a sample of *N* = 714.^3^

The overall model explained 41.1% of the variance in the likelihood of voting for the AfD^4^ and 35.0% of the variance in commitment to societal change. The fit was acceptable: χ^2^(1018) = 5,865.68, *p* < .001; CFI = .89, TLI = .89, RMSEA = .08, 90% CI = [0.08, 0.08], SRMR = .09. Participants in the intervention reported a higher uncertainty-as-enabling mindset than the control group (β = .11, *p* = .021). The intervention had no direct effect on the two diversity measures, perceived productivity (β = –.01, *p* = .708), and aversion (β = .02, *p* = .582), nor the likelihood of voting for the AfD (β = –.05, *p* = .390), but a small negative effect on commitment to societal change (β = –.09, *p* = .011).

Figure 3 presents the fully standardized path coefficients, and Table 3 provides the corresponding unstandardized estimates, standard errors, and 95% CIs. Consistent with H1, a higher uncertainty-as-enabling mindset was associated with (a) greater perceived productivity of diverse groups and (b) lower aversion to intergroup contact.

**Figure 3.**
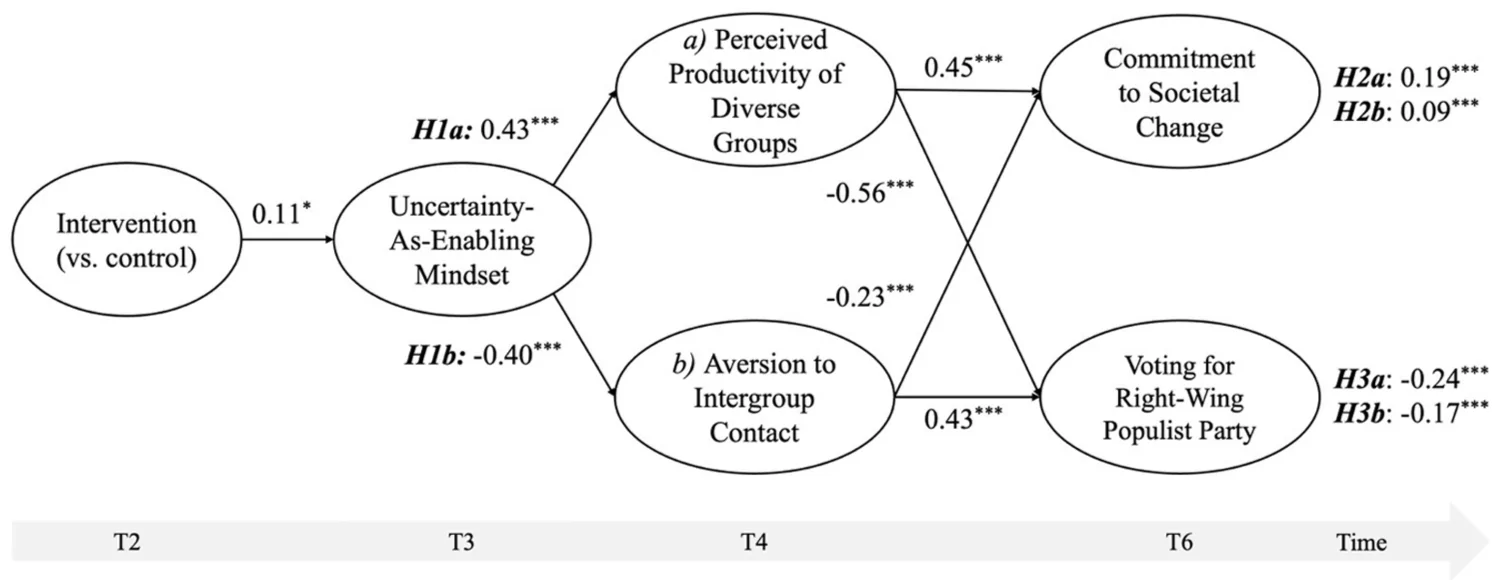
Structural Equation Model Predicting Commitment to Societal Change and Right-Wing Populist Voting Behavior. *Note*. All parameters shown are fully standardized. *p < .05. **p < .01. ***p < .001.

**Table 3:** Unstandardized Estimates, SEs, and Confidence Intervals of the SEM

Parameter	*b*	*SE*	95% CI	*p*	β
**Direct effects**					
H1a (mindset → productivity)	0.74	.11	[0.53, 0.96]	< .001	.43
H1b (mindset → aversion)	–0.96	.16	[–1.27, –0.65]	< .001	–.40
Mindset → change commitment	0.18	.11	[–0.05, 0.40]	.128	.10
Mindset → voting right-wing populism	0.53	.13	[0.18, 0.35]	< .001	.36
Productivity → change commitment	0.48	.05	[0.39, 0.58]	< .001	.45
Productivity → voting right-wing populism	–0.48	.04	[–0.56, –0.39]	< .001	–.56
Aversion → change commitment	–0.18	.04	[–0.25, –0.10]	< .001	–.23
Aversion → voting right-wing populism	0.27	.04	[0.18, 0.35]	< .001	.43
**Indirect effects**					
H2a (mindset → productivity → change commitment)	0.36	.06	[0.24, 0.48]	< .001	.19
H2b (mindset → aversion → change commitment)	0.17	.04	[0.08, 0.26]	< .001	.09
H3a (mindset → productivity → voting right-wing populism)	–0.35	.06	[–0.47, –0.24]	< .001	–.24
H3b (mindset → aversion → voting right-wing populism)	–0.26	.06	[–0.37, –0.14]	< .001	–.17

*Note. N* = 714. *b* = unstandardized coefficient; *SE* = standard error; CI = confidence interval; *p* = *p* value (two-tailed), β = standardized coefficient. Mindset = uncertainty mindset; productivity = perceived productivity of diverse groups; aversion = aversion to intergroup contact; change commitment = commitment to societal change; voting right-wing populism = likelihood of voting for a right-wing populist party (AfD).

Supporting H2, the mindset was indirectly linked to a stronger commitment to societal change via (a) greater perceived productivity and (b) lower aversion. Finally, in line with H3, a higher uncertainty-as-enabling mindset was indirectly linked to a lower likelihood of voting for the AfD through the same pathways of (a) greater perceived productivity and (b) lower aversion.

To assess robustness, we conducted supplementary analyses. First, we reran the main model with the diversity-related measures from T5 (instead of T4), which produced virtually identical estimates and patterns of significance (SM5). Second, we fit a multi-group SEM, separating intervention and control groups (instead of including the intervention variable as a predictor) and found that the hypothesized pathways (H1–H3) held in both conditions (SM6). Third, we added self-reported left–right political orientation (T6) as a covariate^5^ in our main model (SM7), which left the pattern of results largely unchanged. Several paths showed attenuated evidence but remained significant at α = .05. The only substantive change was that the aversion → change commitment path became non-significant (β = .03, *p* = .841), and H2b was no longer supported (β = –.02, *p* = .842).

## Discussion

Uncertainty often carries a negative reputation in the socio-political domain: When experienced as fixed and threatening, it consistently links with ideologies that undermine intergroup relations. Moreover, people may become less open to social change toward equality and more supportive of extremist groups, such as right-wing populist movements. These outcomes threaten democratic values, such as pluralism and the protection of minority rights. Our study aimed to demonstrate that appraising uncertainty as malleable and an opportunity may counteract these adverse effects. By fostering an uncertainty-as-enabling mindset, we sought to strengthen openness to social transformation and lower support for right-wing populist parties.

Employing an uncertainty mindset intervention in a multi-wave experimental study, we show that a 7.5-minute exercise about uncertainty was linked to a higher uncertainty-as-enabling mindsets at 1 week, 2 weeks, and 1 month post-intervention compared with a control group—that is, participants came to view uncertainty as more malleable and an opportunity and less fixed and as a threat. But what does this more positive uncertainty mindset correspond with? In support of our hypotheses, fostering an uncertainty-as-enabling mindset was associated with more positive diversity-related attitudes (i.e., higher perceived productivity of diverse groups and lower aversion to intergroup contact), which were associated with stronger commitment to societal change after the German 2025 federal election, a few weeks later. Furthermore, the mindset was indirectly associated with a lower likelihood of voting for a right-wing populist party through the same pathways. Our participants self-reported their voting behavior following the election, and we compared those who voted for the AfD with those who did not.

Even after accounting for political orientation, the overall pattern of hypothesized pathways remained, though somewhat attenuated (SM7). The one exception was that the indirect path from the uncertainty-as-enabling mindset to commitment to societal change via aversion to intergroup contact was no longer significant. This indicates that the link between aversion and commitment is better explained by political orientation than by aversion itself. However, because political orientation was measured only after the outcomes (SM2), this finding should be interpreted with caution. Taken together, the results provide converging support for our model: Diversity attitudes mediate the relationship between uncertainty mindsets and socio-political consequences. These findings open several avenues for future research on how uncertainty appraisals shape intergroup attitudes and political behavior, and how cultivating an uncertainty-as-enabling mindset might serve as a democratic coping strategy in times of heightened uncertainty.

Our work makes several contributions to the existing literature. First, adopting an uncertainty regulation perspective (Griffin & Grote, 2020), we highlight that an uncertainty-as-enabling mindset is not only relevant for individual self-regulation or work behaviors but also linked to socio-political attitudes and behaviors. Because right-wing populist movements gain traction by exploiting uncertainty and related fear and ambiguity (e.g., Mols & Jetten, 2020), fostering a mindset that views uncertainty as an opportunity and malleable may prove to be a critical resource for strengthening positive attitudes toward diversity, which is linked to a more general commitment to social equality and rejection of extremism, which is what Mudde (2010) defines as “anti-democracy.” This focus is particularly timely, as contemporary democracies are increasingly marked by political instability, demographic change, and institutional volatility, conditions that are both uncertain and subjectively threatening (e.g., Craig & Richeson, 2014; Stollberg & Jonas, 2021; Wohl et al., 2010). The German 2025 federal election period provided a real-world context of upheaval (SM1), where uncertainty was salient and consequential (Kreilinger, 2025; McGuinness, 2024).

To ensure our findings were not driven by support for any specific party or political orientation, we applied our model to predict voting for the center-right Christian Democratic Union (CDU/CSU). As detailed in SM8, the model only explained 0.18% of the variance, and diversity-related attitudes neither predicted support for the CDU/CSU nor mediated the effect of the uncertainty-as-enabling mindset on CDU/CSU voting. Although the CDU/CSU adopted a more socially right-wing approach in the 2025 federal election (Thomeczek et al., 2024), it is not considered a populist party. Moreover, our main findings held even when we combined the AfD with the newly formed left-wing nationalist BSW into a single category, a party that exhibited some characteristics of a populist orientation (Leunig, 2025). These additional results align with our prediction that the identified pathways are specific to right-wing populist support in our context.

Our study also expands Griffin and Grote’s (2020) uncertainty regulation theory by introducing evidence from a social psychology perspective and looking beyond intraindividual processes to encompass behaviors and outcomes at both interpersonal and intergroup levels. Whereas Hogg’s (2007, 2014) uncertainty–identity theory explains why uncertainty can drive extreme political behaviors (namely, the motive to *reduce* uncertainty), our approach reconceives cognitive and emotional flexibility to uncertainty not as a trait or an inevitable casualty in times of uncertainty but as a controllable product of individual appraisal. Indeed, our findings seem to indicate that when people appraise uncertainty as malleable and an opportunity, they engage in responses reflecting exploration tendencies even in intergroup contexts rather than retreating into ideological rigidity (Halperin et al., 2012). Thus, the uncertainty mindset may reflect a form of cognitive flexibility that predicts extreme political orientations (Zmigrod et al., 2019).

Our second contribution lies in providing initial evidence that more positive attitudes toward diverse groups can be created indirectly through interventions targeting an uncertainty-as-enabling mindset, rather than by directly confronting issues of diversity and prejudice. Traditionally, prejudice-reduction strategies rely on direct exposure to outgroup members or politically sensitive content (Čehajić-Clancy & Halperin, 2024), which can evoke resistance, especially among individuals with extreme attitudes (e.g., Harris & Van Bavel, 2021), and demand characteristics (Paluck et al., 2021). Our findings suggest that reframing the broader contextual lens through which individuals interpret uncertainty can have a positive influence on attitudes toward diversity. Thus, cultivating a mindset that embraces the uncertainty inherent in everyday life may prime individuals for greater receptivity to diversity, even without references to politically charged topics. Together, our results bridge different streams of research and underscore the broader implications of uncertainty mindsets for understanding and influencing political behavior.

However, two alternative explanations merit consideration. First, could a greater comfort with uncertainty also fuel support for unconventional solutions, including right-wing populism? One might argue that an uncertainty-as-enabling mindset could reflect a general appetite for “doing something different,” which may include backing fringe parties. In contrast, our data show that participants higher in an uncertainty-as-enabling mindset reported lower support for a party we classified as right-wing populist based on existing literature (e.g., Mudde, 2010, 2019). This finding may be linked to a core feature of right-wing populism: It is not fundamentally a vehicle for social change, but rather a defense of exclusionary, anti-diversity norms and policies (e.g., Meléndez & Kaltwasser, 2023; Mudde, 2007). That said, we acknowledge the nuance in our commitment to societal change scale, which was adapted from Herscovitch and Meyer (2002). The scale was worded neutrally to minimize participant attrition or signal an ideological norm. In theory, this scale could have captured a generic desire to overturn any status quo, including support for right-wing or populist alternatives to mainstream government (Becker, 2020). However, in our data, commitment to change aligned positively with support for the political system, democratic values, and left-leaning political orientation (SM3). Thus, we interpret it as a proxy for support of progressive rather than reactionary societal change.

Another consideration is whether self-report biases could underlie our behavioral findings. As with any survey relying on participants’ own accounts, we cannot fully exclude the possibility of misrepresentation. Yet several factors argue against an explanation that participants may have been untruthful. First, the AfD now holds the second-largest share of seats in the German parliament (The Federal Returning Officer, 2025), so endorsing the AfD is not necessarily socially stigmatized. Second, although 30 participants selected “prefer not to say,” this refusal rate is modest. While it is conceivable that these non-responders disproportionately support the AfD or a smaller (right-wing) populist-oriented party, there is no straightforward way to ascertain or correct for that pattern.^6^ While it is impossible to rule out all self-report biases, the convergence between our survey data and real-world election outcomes provides some reassurance.

### Strengths, Limitations, and Future Directions

Our study has several key strengths and limitations. First, our study is limited by the psychometric properties of the scales used to assess participants’ attitudes. While each scale performed well individually, the joint measurement model showed acceptable, but not optimal fit (Table 3). One possible explanation is method bias because the use of the same Likert-type response format across constructs may have introduced shared method variance (Podsakoff et al., 2012).

Second, by fielding it during Germany’s federal election, marked by high uncertainty and tangible political consequences (votes and electoral outcomes), we achieved high ecological validity for our research question. At the same time, participants were recruited from an opt-in online panel. Although age- and gender-based quotas were applied at T1 to approximate the German population, and the sample’s reported voting behavior closely matched the actual electoral results (Table 1), it is not representative, and inferences should be interpreted accordingly. Moreover, our focus on German citizens and politics constrains cross-national generalizability (see Uskul et al., 2024). Although political trust in Germany is comparable with the OECD average (Organization for Economic Co-operation and Development [OECD], 2024), its institutional and political context differs from many other settings. Germany also scores relatively high on uncertainty avoidance, with citizens rather uncomfortable with uncertainty (Hofstede et al., 2010). The effects of our mindset intervention may vary in cultures lower or even higher in uncertainty avoidance. Our study, therefore, serves as an initial step that should be replicated across countries and institutional contexts to assess robustness and boundary conditions.

Third, we introduce a novel, mindset-based approach to improving diversity attitudes and intergroup relations. Our wise intervention approach teaches people to regulate uncertainty, rather than minimize or avoid it. This approach is brief, easy to deliver, and effective, and by focusing on uncertainty rather than diversity directly, it sidesteps common pitfalls, such as reactance, dropout, anger, or demand characteristics. However, its effect size was modest for practical impact. Prior work assessed mindset shifts immediately after the exercise (Strittmatter et al., 2024), whereas we measured them one to multiple weeks later. The fact that our effects persisted across this time period is encouraging, but we acknowledge that larger effects would enhance practical impact.

Finally, future research could examine the effectiveness of the uncertainty mindset intervention on validated measures of right-wing populist attitudes (e.g., Jami & Kemmelmeier, 2025). Considering that voting behavior is often influenced by non-ideological factors (Schoen et al., 2017), which is also reflected in our non-significant association between political orientation and right-wing populist voting (see SM7), such measures would allow assessment of whether the intervention shifts beliefs. This approach would also clarify for whom the intervention is most effective and how it might be tailored to different populations. For example, less ideologically committed individuals may be more receptive to the intervention than those with strong right-wing populist beliefs.

Taken together, our study provides a promising foundation for understanding and harnessing uncertainty mindsets in the socio-political context. Going forward, researchers could develop and evaluate more intensive uncertainty mindset interventions to amplify effect sizes, replicate these interventions across diverse political systems and cultural settings to assess generalizability, and incorporate a broader set of political measures. Future work would also benefit from including objective indicators of diversity-related and political behaviors, rather than relying solely on self-reports. By addressing these avenues, future research can both strengthen the practical impact of uncertainty mindset interventions and deepen our insights into their real-world application.

## Conclusion

Against the backdrop of Germany’s 2025 early federal elections, called amid heightened political and social uncertainty, we tested an uncertainty mindset intervention to determine whether fostering an uncertainty-as-enabling mindset would promote more positive diversity attitudes, which in turn would increase commitment to societal change and reduce support for right-wing populist parties. The substantial gains of right-wing populist parties (e.g., the AfD received 69 seats in the government, making it the second-largest party) show that it may be imperative to identify strategies to help citizens navigate uncertainty without undermining social cohesion or democratic values. Our short mindset exercise successfully prompted participants to view uncertainty as both malleable and an opportunity. This mindset predicted more inclusive intergroup attitudes, which were associated with stronger commitment to social transformation and reduced voting for the AfD. These results highlight the promise of framing uncertainty as a resource for enhancing openness to diversity and resisting exclusionary political appeals. Overall, this work lays the groundwork for using uncertainty mindset interventions to foster social cohesion when political and social instability heightens people’s sense of threat.

## Supplemental Material

sj-docx-1-psp-10.1177_01461672251386487 – Supplemental material for The Role of Uncertainty Mindsets in Shaping Diversity Attitudes and Their Downstream Effects on Commitment to Societal Change and Right-Wing Populist Voting Supplemental material, sj-docx-1-psp-10.1177_01461672251386487 for The Role of Uncertainty Mindsets in Shaping Diversity Attitudes and Their Downstream Effects on Commitment to Societal Change and Right-Wing Populist Voting by Ruri Takizawa, Stefanie Marx-Fleck, Alina Gerlach and Gudela Grote in Personality and Social Psychology Bulletin

## Data Availability

The data, code, codebook, and materials are available at http://osf.io/9ks2n.

## Notes

1. https://schoemanna.shinyapps.io/mc_power_med/.

2. We assessed non-binary gender and offered a non-response option, but after data cleaning, only participants who self-identified as men or women remained.

3. We conducted a Monte Carlo simulation to estimate power for commitment to societal change (Schoemann et al., 2017). With 1,000 replications, 10,000 bootstrap draws, and α = .05, *N* = 714 yielded power = 1.00 for the indirect path via perceived productivity, .73 for the path via aversion to intergroup contact, and 1.00 for their difference.

4. We also ran a supplementary analysis in which we combined votes for the parties Alternative for Germany (*Alternative für Deutschland*, AfD) and Bündnis Sahra Wagenknecht (BSW) (rather than AfD alone) based on their similar political orientation on social and immigration-related issues and populist rhetoric. The pattern of results remained unchanged, although the paths had smaller effect sizes. The model accounted for 28.9% of the variance in voting for AfD or BSW (vs. other behaviors).

5. Because we aimed to avoid signaling that the study concerned politics (which could have increased attrition and demand characteristics), we measured political orientation (Kroh, 2007) only at T6, after the two primary outcomes. As a post-treatment measure, political orientation is not independent of the intervention and thus has limited suitability as a covariate. Accordingly, we report the analysis in the Supplemental Material.

6. We conducted a logistic regression predicting “Do not want to indicate” (1 = *did not indicate*; 0 = *indicated behavior at the election*) using demographic variables, measures from the main analyses, and additional political variables from T6. Only support for democratic values predicted non-disclosure of voting behavior: individuals with lower support for democratic values were more likely not to report their behavior (β = .51, 95% CI = [0.32, 0.82], *p* = .005).
