# Supplementary material for: The Role of Uncertainty Mindsets in Shaping Diversity Attitudes and Their Downstream Effects on Commitment to Societal Change and Right-Wing Populist Voting
Source: Pers Soc Psychol Bull. 2025 Nov 11;52(11):3673–87. doi: 10.1177/01461672251386487 (PMC13646405; doi:10.1177/01461672251386487)
Supplement: Supplementary file 1 [file sj-docx-1-psp-10.1177_01461672251386487.docx]

**Supplemental Materials (SM) for “The Role of Uncertainty Mindsets in Shaping Diversity Attitudes and Their Downstream Effects on Commitment to Societal Change and Right-Wing Populist Voting”**

Table of Contents

[SM 1. Timeline of Germany’s Domestic News Topics During the Data Collection Period, with Emphasis on Election-Related Issues and Immigration or Refugee Matters	2](#_Toc206514421)

[2024	2](#_Toc206514422)

[2025	2](#_Toc206514423)

[SM 2. Additional Measures	5](#_Toc206514424)

[SM 3. Correlations between Commitment to Societal Change and Other Measures at T6	8](#_Toc206514425)

[SM 4. CFA with Diversity Attitude Measures from T5.	9](#_Toc206514426)

[SM 5. Hypothesis-Testing SEM with Diversity Attitudes from T5.	10](#_Toc206514427)

[SM 6. Multi-Group SEM	11](#_Toc206514428)

[SM 7. Hypothesis-Testing SEM with Political Orientation as a Covariate	12](#_Toc206514429)

[SM 8. SEM on Likelihood of Voting for CDU/CSU.	14](#_Toc206514430)

## SM 1. ****Timeline of Germany’s Domestic News Topics During the Data Collection Period, with Emphasis on Election-Related Issues and Immigration or Refugee Matters****

### 2024

**16.12**: The 205th session of the current Bundestag cleared the way for new elections. As planned, Chancellor Olaf Scholz lost the vote of confidence and asked President Steinmeier to dissolve the Bundestag ([Bundeskanzler Olaf Scholz verliert die Vertrauensfrage)](https://www.tagesschau.de/inland/scholz-verliert-vertrauensfrage-100.html). Following the collapse of the “Ampel” coalition, Scholz thus paved the way for early elections on February 23.

**20.12**: A driver plowed into a crowd of people at the Christmas market in downtown Magdeburg. Five people were killed, and more than 200 were injured ([Der Weihnachtsmarkt-Anschlag und seine Folgen](https://www.tagesschau.de/inland/innenpolitik/magdeburg-faq-104.html)).

**31.12:** New Year’s Eve accidents and assaults ([Silvesterunfälle und Angriffe](https://www.welt.de/vermischtes/article255000750/Berlin-26-Jaehriger-kritisiert-Maenner-fuers-Boellern-und-wird-ins-Gleisbett-gestossen.html)).

### 2025

**22.01:** Two people (a child and an adult) were killed in a knife attack in a park in Aschaffenburg ([Messerangriff in Aschaffenburg](https://www.tagesschau.de/inland/gesellschaft/aschaffenburg-messerangriff-100.html)).

**25.01:** Protests in Berlin against the AfD, right-wing parties in Europe, politics under Donald Trump, and Elon Musk’s influence (with some posters also targeting CDU leader Merz). Demonstrations also took place in Aschaffenburg and other cities ([Demos gegen Rechtsdruck](https://www.tagesschau.de/inland/demos-gegen-rechtsruck-102.html)).

**26.01**: In the dispute over migration policies, the CDU/CSU faction specified its ideas and formulated two motions for the Bundestag, in which they demand permanent border controls and the rejection of all attempts to enter the country illegally without exception ([Unionsanträge zur Migration](https://www.tagesschau.de/inland/innenpolitik/unionsfraktion-antraege-migrationspolitik-100.html)).

**29.01:** Hundreds of people demonstrate in front of the CDU party headquarters in Berlin against the joint vote by the CDU/CSU and AfD ([Demo vor der CDU-Parteizentrale](https://www.tagesschau.de/newsticker/liveblog-bundestagswahl-mittwoch-104.html)).

**05.02**: The leading politicians from the AfD, FDP, BSW, Left, Greens, and CSU debated two key campaign issues (migration and the economy) on the ZDF program “Schlagabtausch” ([ZDF-Schlagabtausch](https://www.zdf.de/video/magazine/zdfheute-live-102/politiker-schlagabtausch-wahl-video-100)).

**09.02**: Scholz (SPD) and Merz (CDU/CSU) debated for 90 minutes on TV (head-to-head debate between the SPD and CDU/CSU chancellor candidates; [TV Duell](https://www.tagesschau.de/inland/bundestagswahl/parteien/tv-duell-scholz-merz-100.html)).

**13.02**: The trial for the Mannheim market-square attack (May 2024), in which a police officer was killed, has begun in Stuttgart ([Prozessauftakt](https://www.tagesschau.de/inland/gesellschaft/prozess-messerangriff-mannheim-100.html)).

**13.02:** The four chancellor candidates (Scholz, Habeck, Merz, and Weidel) appeared as guests on the ZDF program “[Klartext](https://www.zdfheute.de/politik/deutschland/bundestagswahl-klartext-scholz-habeck-weidel-merz-100.html)” (they were questioned one after the other by members of the audience).

**13.02**: Attack on a demonstration in Munich: a two-year-old child and their mother were killed. [A man](https://www.zdfheute.de/politik/deutschland/muenchen-tatverdaechtiger-ausreisepflicht-100.html) drove his car into a demonstration organized by the ver.di trade union ([Anschlag in München](https://www.tagesschau.de/inland/muenchen-anschlag-tote-100.html)).

**14.02:** Thousands of people took to the streets in Berlin, Hamburg, and other cities to demand stronger climate protection ([“Fridays for Future”](https://www.tagesschau.de/inland/gesellschaft/klimaschutz-demo-108.html)).

**15.02**: Christopher Street Day events took place in approximately 50 locations across Germany, sending a message of diversity, inclusion, and opposition to hate ([Wähl Liebe](https://www.waehl-liebe.de/)).

**16.02**: Four-way round of the chancellor candidates. It was their first time debating as a group of four ([Quadrell](https://www.tagesschau.de/multimedia/video/video-1434618.html)).

**19.02:** Commemoration for the victims of Hanau (five years since the attack). President Steinmeier calls for unity ([Zusammenhalt)](https://www.tagesschau.de/inland/hanau-gedenken-steinmeier-100.html).

**23.02:** Federal elections (voter turnout at 82.5%) and initial results. Merz announces that he aims to complete the formation of a government by Easter ([Ergebnisse](https://www.tagesschau.de/wahl/archiv/2025-02-23-BT-DE/index.shtml)).

**24.02**: The results of the federal elections became clearer. Merz announces talks with the SPD ([Ergebnisse)](https://www.tagesschau.de/newsticker/liveblog-bundestagswahl-2025-montag-100.html).

**25.02:** Most parliamentary groups in the Bundestag held meetings, attended by both outgoing and newly elected members.

**26.02:** The new SPD parliamentary group in the Bundestag elected party leader Klingbeil as chairman.

**28.02:** First talks between the CDU/CSU and SPD.

**02.03:** Hamburg parliamentary elections 2025 ([Bürgerschaftswahl](https://www.tagesschau.de/wahl/archiv/2025-03-02-LT-DE-HH/index.shtml)).

## SM 2. Additional Measures

The measures are presented in the order they appeared on the questionnaires. Seven-point Likert scales were used unless otherwise indicated. If original scales were only available in English, we conducted a back-translation procedure into German.

***Diversifying Information Sources (T3-5)***

We adapted the information-seeking scale from Morrison (1993), originally developed in an organizational context, to a broader setting by asking participants about their behavior over the past seven days. Ten items assessed how frequently participants engaged in diverse information-seeking behaviors (*never* to *very often*), including items such as “engaged with someone outside your usual social circle to gain a fresh perspective.” The scale demonstrated high internal consistency (Cronbach’s α_T3-5_ = .92-.94).

***Benefits and Threats of Cultural Diversity (T3-5)***

We used Hofhuis et al.’s (2015) scale to assess whether individuals perceived cultural diversity as a benefit or a threat. To reduce questionnaire length, we selected only the dimensions deemed most relevant. This resulted in 12 items measuring perceived benefits (from the categories: understanding diverse groups, creative potential, social environment) and 12 items measuring perceived threats (from the categories: realistic threat, symbolic threat, intergroup anxiety). Composite scores were calculated for each time point, with high internal consistency for both perceived benefits (αₜ₃–ₜ₅ = .97) and perceived threats (αₜ₃–ₜ₅ = .94).

***Attitudes Toward Diverse Groups in Society (T3)***

We reported the descriptive statistics for T4 and T5 in the manuscript. Diversity-related attitudes were also assessed at T3: Perceived productivity of diverse groups (α = .94, M = 4.62, SD = 1.20) and aversion to intergroup contact (α = .84, M = 4.11, SD = 1.20).

***Satisfaction with the Political System (T6)***

We measured participants’ satisfaction with the political system with the German short scale by Dentler et al. (2020), which comprises four items (α = .81, *M* = 4.12, *SD* = 1.36). An example item is: “The political system in Germany is just and fair.”

***Support for Democratic Values (T6)***

Six items based on Canetti-Nisim (2004) assessed support for democratic values (see also Hebel-Sela et al., 2024), with items like “Germany must provide equal social and political rights to all citizens, regardless of religion, race, or gender” (α = .63, *M* = 5.19, *SD* = 0.94).

***Party Identity Strength (T6)***

Participants, who indicated which party they voted for in the election, were subsequently asked to what extent they identified with that party (1 item; n = 684, M = 5.24, SD = 1.30).

***Post-Election Anxiety (T6)***

Based on Danbold et al. (2023), we measured participants’ emotions regarding the election outcome (*fearful*, *anxious*, *optimistic*). To balance the scale’s valence, we added a fourth item (*hopeful*). The positive items were reverse-coded to create a composite score of post-election anxiety (α = .84, *M* = 4.41, *SD* = 1.39).

***Past Voting Behavior (2021; T6)***

We asked participants about their voting behavior in the 2021 federal election and whether it was consistent with their recent behavior, i.e., whether they voted for the same party or did not vote again (51.1%), whether they behaved differently (e.g., voted for a different party; 41.2%), or whether they did not wish to indicate or could not recall their previous behavior (7.4%).

***Political Orientation (T6)***

One item assessed participants’ general political orientation from 1 *very left* to 11 *very right* (Kroh, 2007; *M* = 6.00, *SD* = 1.90).

***National Identification (T6)***

Participants’ national identification was measured with three items (α = .89, *M* = 4.94, *SD* = 1.53) like “being German is an important part of who I am” (e.g., Martinovic & Verkuyten, 2012).

***Voice Behavior (T6)***

Finally, six items adapted from LePine and Van Dyne (1998) were used to measure participants’ voice behavior regarding political issues (α = .91, *M* = 3.88, *SD* = 1.52). An example item is: “I communicate my opinion on political issues to others, even when my views differ and the majority disagrees”.

## SM 3. Correlations between Commitment to Societal Change and Other Measures at T6

Commitment to societal change was significantly, though weakly, correlated with other measures assessed at T6. It was positively associated with support for the political system (r = .36, p < .001), support for democratic values (r = .32, p < .001), and voice behavior (r = .11, p = .002). It was negatively associated with post-election anxiety (r = -.15, p < .001), right-wing political orientation (r = -.27, p < .001), and national identification (r = -.13, p < .001).

## SM 4. CFA with Diversity Attitude Measures from T5.

The four‐factor model fit well, and outperformed the three‐factor model, Δχ²(238) = 672.34, *p* < .001, and the one‐factor model, Δχ²(10) = 10638.68, *p* < .001 (see Table 1).

**Table 1**

*Configurations with Diversity-Related Attitudes Measured at T5.*

|  | **χ²(df), *p*** | **CFI** | **TLI** | **RMSEA [90% CI]** | **SRMR** |
| --- | --- | --- | --- | --- | --- |
| Four Factors | χ²(935) = 3044.28, *p* < .001 | .90 | .90 | .06 [.05, .06] | .08 |
| Three Factors | χ²(697) = 3716.61, *p* < .001 | .84 | .83 | .08 [.07, .08] | .09 |
| One Factor | χ²(945) = 13682.96, *p* < .001 | .42 | .40 | .14 [.13, .14] | .16 |

## SM 5. Hypothesis-Testing SEM with Diversity Attitudes from T5.

The main analysis reported in the manuscript was repeated with the diversity attitudes (perceived productivity of diverse groups and aversion to intergroup contact) measured at T5 (instead of T4). The overall model with 705 used cases explained 36.8% of the variance in the likelihood of voting for a right-wing populist party and 37.2% of the variance in commitment to societal change, χ²(1018) = 3677.78, p < .001; CFI = .89, TLI = .88; RMSEA = .08, 90% CI [.08, .09]; SRMR = .09 (see Figure 1 for the pathways).

**Figure 1**

*Structural equation model (T3→T5*→*T6) predicting commitment to societal change and right-wing populist voting behavior.*

*Note*. All parameters shown are fully standardized.

^*^ = *p* < .05, ^**^ = *p* < .01, ^***^ = *p* < .001.

## SM 6. Multi-Group SEM

We fitted the *Mindset (T3) → Diversity Attitudes (T4) → Outcome* *(T6)* model as a two-group SEM (intervention vs. control). We first compared a version with freely estimated latent intercepts for the uncertainty mindset to one with those intercepts constrained equal. Because the two models did not differ significantly, χ²(37) = 40.21, p = .330, we retained the more parsimonious, intercept-invariant solution: χ²(1954) = 6484.59, CFI/TLI ≈ .90/.89, RMSEA = .08, 90% CI [.08, .08], SRMR = .10. This model accounted for 34.6% (control) and 47.7% (intervention) of the variance in the likelihood of voting for a right-wing populist party (the AfD), and 35.2% (control) and 35.3% (intervention) of the variance in commitment to societal change. Standardized path coefficients for both groups are displayed in Figure 2.

**Figure 2**

*Two-group (intervention vs control) structural equation model (T3→T4*→*T6) predicting commitment to societal change and right-wing populist voting behavior.*

*Note*. All parameters shown are fully standardized.

^*^ = *p* < .05, ^**^ = *p* < .01, ^***^ = *p* < .001.

## SM 7. Hypothesis-Testing SEM with Political Orientation as a Covariate

We incorporated political orientation at T6 (centered; higher scores = more right-leaning) as an exogenous covariate: It was included as a predictor of both T6 outcomes, and we allowed it to covary with the intervention and upstream constructs (uncertainty-as-enabling mindset, perceived productivity, and aversion) to mitigate post-treatment adjustment bias. The extended model showed acceptable fit, χ²(1059) = 5338.44, CFI = .91, TLI = .90, RMSEA = .08, SRMR = .08, and explained 56.6% of the variance in right-wing populist voting and 34.2% in commitment to societal change. Political orientation was not significantly related to right-wing populist voting (β = -.05, *p* = .844). Left-leaning political orientation was associated with commitment to societal change (β = -.22, *p* = .029). Results are reported in Figure 3 and Table 2. Overall, the point estimates were similar to the main model without the covariate. For some paths, p-values were larger, while remaining significant at α = .05. A substantive change was that the Aversion → Change commitment path became non-significant, and H2b was no longer supported.

**Figure 3**

*Structural Equation Model Predicting Commitment to Societal Change and Right-Wing Populist Voting Behavior While Controlling for Political Orientation.*

*Note*. All parameters shown are fully standardized.

^*^ = *p* < .05, ^**^ = *p* < .01, ^***^ = *p* < .001.

**Table 2**

*Unstandardized Estimates, SEs, and Confidence Intervals of the SEM Model Controlling for Political Orientation.*

| **Parameter** | ***b*** | **SE** | **95% CI** | ***p*** | **β** |
| --- | --- | --- | --- | --- | --- |
| **Direct effects** |  |  |  |  |  |
| H1a (Mindset → Productivity) | 1.10 | .15 | [0.80, 1.39] | <.001 | .64 |
| H1b (Mindset → Aversion) | -1.56 | .25 | [-2.04, -1.07] | <.001 | -.64 |
| Mindset → Change commitment | 0.69 | .39 | [-0.07, 1.44] | .076 | .37 |
| Mindset → Voting right-wing populism | 1.26 | .61 | [0.06, 2.46] | .040 | .85 |
| Productivity → Change commitment | 0.30 | .12 | [0.07, 0.53] | .011 | .28 |
| Productivity → Voting right-wing populism | -0.62 | .17 | [-0.95, -0.28] | <.001 | -.72 |
| Aversion → Change commitment | 0.02 | .11 | [-0.19, 0.23] | .841 | .03 |
| Aversion → Voting right-wing populism | 0.42 | .18 | [0.06, 0.78] | .022 | .70 |
| **Indirect effects** |  |  |  |  |  |
| H2a (Mindset → Productivity → Change commitment) | 0.33 | .13 | [0.07, 0.58] | .013 | .18 |
| H2b (Mindset → Aversion → Change commitment) | -0.03 | .17 | [-0.36, 0.30] | .842 | -.02 |
| H3a (Mindset → Productivity → Voting right-wing populism) | -0.67 | .22 | [-1.10, -0.25] | .002 | -.46 |
| H3b (Mindset → Aversion → Voting right-wing populism) | -0.65 | .33 | [-1.29, -0.01] | .045 | -.44 |

*Note.* N = 714. *b* = unstandardized coefficient; SE = standard error; CI = 95% confidence intervals; *p* = p-value (two-tailed), β = standardized coefficient. Mindset = uncertainty mindset; productivity = perceived productivity of diverse groups; aversion = aversion to intergroup contact; change commitment = commitment to societal change; voting right-wing populism = likelihood of voting for a right-wing populist party (AfD).

## SM 8. SEM on Likelihood of Voting for CDU/CSU.

The main analysis reported in the manuscript, which used diversity-related measures from T4, was repeated using a different categorization of the voting behavior dependent variable (see Figure 4). Instead of examining the likelihood of voting for the AfD versus other behaviors, we analyzed the likelihood of voting for the Christian Democrats (CDU/CSU) versus all other behaviors. The overall model, based on 714 used cases, explained 0.2% of the variance in the likelihood of voting for the CDU/CSU and 3.0% of the variance in commitment to societal change, χ²(1018) = 5810.45, p < .001; CFI = .89, TLI = .89; RMSEA = .08, 90% CI [.08, .08]; SRMR = .09.

**Figure 4**

*Structural equation model (T3→T4*→*T6) predicting commitment to societal change and CDU/CSU voting behavior.*

 *Note*. All parameters shown are fully standardized.

^*^ = *p* < .05, ^**^ = *p* < .01, ^***^ = *p* < .001.

# References of the SM

Canetti-Nisim, D. (2004). The Effect of Religiosity on Endorsement of Democratic Values: The Mediating Influence of Authoritarianism. *Political Behavior, 26,* 377–398. <https://doi.org/10.1007/s11109-004-0901-3>

Danbold, F., Serrano-Careaga, J., & Huo, Y. J. (2023). Prototypicality threat drives support for nativist politics in US and UK elections. *Current Research in Ecological and Social Psychology*, *4*, 100080. <https://doi.org/10.1016/j.cresp.2022.100080>

Dentler, K., Bluemke, M., & Gabriel, O.W. (2020). German Satisfaction with the Political System Short Scale (SPS).

*Zusammenstellung sozialwissenschaftlicher Items und Skalen (ZIS).*

<https://doi.org/10.6102/zis278>

Hebel‐Sela, S., Aldar, L., Orian Harel, T., Hameiri, B., Pliskin, R., & Halperin, E. (2024). Gone too far? The paradoxical effect of political elite radicalization. *Political Psychology. Online Version.* <https://doi.org/10.1111/pops.13068>

Hofhuis, J., van der Zee, K. I., & Otten, S. (2015). Measuring employee perception on the effects of cultural diversity at work: Development of the benefits and threats of diversity scale. *Quality & Quantity, 49*, 177-201. <https://doi.org/10.1007/s11135-013-9981-7>

Kroh, M. (2007). Measuring left–right political orientation: The choice of response format. *Public Opinion Quarterly*, *71*(2), 204-220. [https://doi.org/10.1093/poq/nfm009](https://psycnet.apa.org/doi/10.1093/poq/nfm009)

LePine, J. A., & Van Dyne, L. (1998). Predicting voice behavior in work groups. Journal of Applied Psychology, 83(6), 853–868. [https://doi.org/10.1037/0021-9010.83.6.853](https://psycnet.apa.org/doi/10.1037/0021-9010.83.6.853)

Martinovic, B., & Verkuyten, M. (2012). Host national and religious identification among Turkish Muslims in Western Europe: The role of ingroup norms, perceived discrimination and value incompatibility. *European Journal of Social Psychology*, *42*(7), 893-903. <https://doi.org/10.1002/ejsp.1900>

Morrison, E. W. (1993). Longitudinal study of the effects of information seeking on newcomer socialization. Journal of Applied Psychology, 78(2), 173-183. <https://doi.org/10.1037/0021-9010.78.2.173>
